# *Bidens pilosa* Extract Administered after Symptom Onset Attenuates Glial Activation, Improves Motor Performance, and Prolongs Survival in a Mouse Model of Amyotrophic Lateral Sclerosis

**DOI:** 10.1155/2020/1020673

**Published:** 2020-01-29

**Authors:** Yasuhiro Kosuge, Erina Kaneko, Hiroshi Nango, Hiroko Miyagishi, Kumiko Ishige, Yoshihisa Ito

**Affiliations:** Laboratory of Pharmacology, School of Pharmacy, Nihon University, 7-7-1 Narashinodai, Funabashi-shi, Chiba 274-8555, Japan

## Abstract

Amyotrophic lateral sclerosis (ALS) is a late-onset neurodegenerative disorder characterized by progressive paralysis resulting from the death of upper and lower motor neurons. There is currently no effective pharmacological treatment for ALS, and the two approved drugs riluzole and edaravone have limited effects on the symptoms and only slightly prolong the life of patients. Therefore, the development of effective therapeutic strategies is of paramount importance. In this study, we investigated whether Miyako Island *Bidens pilosa* (MBP) can alleviate the neurological deterioration observed in a superoxide dismutase-1 G93A mutant transgenic mouse (G93A mouse) model of ALS. We orally administered 2 g/kg/day of MBP to G93A mice at the onset of symptoms of neurodegeneration (15 weeks old) until death. Treatment with MBP markedly prolonged the life of ALS model mice by approximately 20 days compared to that of vehicle-treated ALS model mice and significantly improved motor performance. MBP treatment prevented the reduction in SMI32 expression, a neuronal marker protein, and attenuated astrocyte (detected by GFAP) and microglia (detected by Iba-1) activation in the spinal cord of G93A mice at the end stage of the disease (18 weeks old). Our results indicate that MBP administered after the onset of ALS symptoms suppressed the inflammatory activation of microglia and astrocytes in the spinal cord of the G93A ALS model mice, thus improving their quality of life. MBP may be a potential therapeutic agent for ALS.

## 1. Introduction

Amyotrophic lateral sclerosis (ALS), also known as Lou Gehrig's disease, is a fatal neurodegenerative disease characterized by progressive paralysis due to motor neuron degeneration. Most ALS cases are sporadic, and the cause of sporadic ALS remains largely unknown. Familial ALS (fALS) accounts for the remaining 5 to 10 percent of all ALS cases, and only 20% of fALS cases are linked to a mutation in the gene encoding copper-zinc superoxide dismutase (*SOD1*) [1]. Several transgenic mouse models that carry the mutations found in fALS patients have been generated. Among these, the most widely used model is a transgenic mouse that overexpresses a human *SOD1* transgene with a pathogenic glycine to alanine substitution at the 93^rd^ codon (*SOD1^G93A^*). Overexpression of the mutant *SOD1^G93A^* gene in transgenic mice (G93A mice) results in a progressive paralytic disease in which the clinical features resemble that of ALS in humans [2]. Recently, many new ALS-causing gene defects have been identified, including mutations in the gene encoding fused in sarcoma (*FUS*), TAR DNA-binding protein (*TARDBP*), optineurin (*OPTN*), and *C9ORF72* [3]. However, G93A mice have been regarded as the standard model for the evaluation of therapeutic effects during preclinical studies.

Although the pathogenesis of ALS is extremely intricate and remains largely unknown, inflammation and oxidative stress play a pivotal role in ALS pathogenesis and contribute to the vicious cycle of neurodegeneration in the lumbar spinal cord. There is growing evidence that activated microglia and reactive astrocytes increase in the spinal cord of ALS patients [4] and model mice [5]. Activation of glial cells in ALS is marked by the elevated production of neurotoxic mediators such as reactive oxygen species (ROS), proinflammatory cytokines, and inflammatory mediators [6]. Moreover, astrocytes and microglia are associated with noncell autonomous motor neuronal damage and cell death in ALS [7]. Therefore, to identify effective neuroprotective therapeutic agents for the treatment of ALS, not only the motor neuron but also the neighbouring nonmotor neuron cells including microglia, astrocytes, and blood capillaries require analysis. Several therapeutic agents have been found to delay the onset of disease and prolong the disease course in the ALS patients and model mice. Riluzole and edaravone were successfully transferred into clinical practice. Unfortunately, riluzole prolongs life by only a few months [8] and edaravone only slightly improves patient functionality scores only slightly in a subset of patients [9]. Therefore, the development for more promising disease-modifying therapy for ALS remains urgent.

*Bidens pilosa* L. var. *radiata* SCHERFF (BP) is a species of flowering plant from the Asteraceae family and is an annual weed widely distributed in the tropical and subtropical regions of the world such as Africa, America, China, and Japan. BP is a rich source of phytochemicals including flavonoids and polyynes and has therefore been used in traditional medicine for the treatment of various diseases due to its antioxidant, anti-inflammatory, anticancer, antidiabetic, and antihyperglycemic properties [10]. A variety of BP that is cultivated without agricultural chemicals on the Miyako Islands of Okinawa Prefecture, Japan, is referred to as Miyako Island *Bidens pilosa* L. var. *radiata* SCHERFF (MBP). Caffeic acid, six kinds of chlorogenic acids (neochlorogenic acid, chlorogenic acid, 4-*O*-caffeoylquinic acid, 3,4-di-*O*-caffeoylquinic acid, 3,5-di-*O*-caffeoylquinic acid, and 4,5-di-*O*-caffeoylquinic acid), and seven kinds of flavonoids (rutin, quercetin, quercetin derivatives, hyperin, isoquercitrin, centaurein, and jacein) have been isolated and characterized from MBP using high-performance liquid chromatography (HPLC) analysis [11, 12]. Importantly, MBP has been reported to possess antioxidant, anti-inflammatory, antiallergy, antivirus, and antileukaemia properties [12–16]. Although the diverse phytochemicals and bioactivity of MBP may be useful for the treatment of certain neurodegenerative diseases including ALS, the therapeutic potential of MBP in the treatment of neurodegenerative disorders is still unclear. Therefore, in this study, we evaluated the therapeutic potential of MBP and examined whether MBP could effectively protect neurons and suppress glial activation in the spinal cords in G93A mice.

## 2. Materials and Methods

### 2.1. Animals

**Figure 1:**
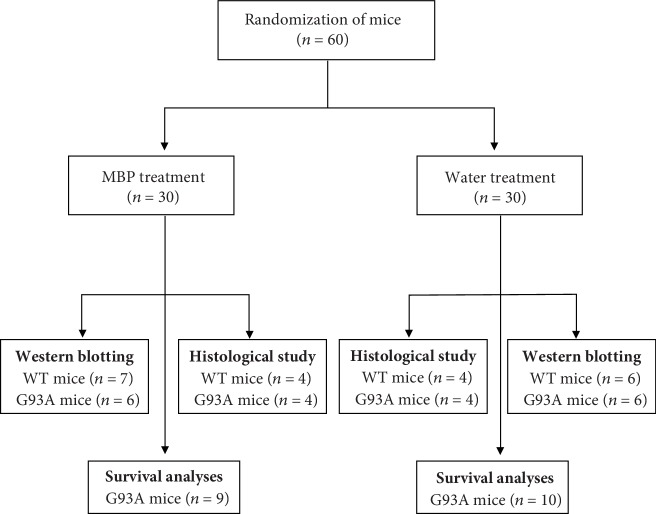
Flowchart of the experiment design. MBP: Miyako Island *Bidens pilosa* var. *radiata* SCHERFF.

G93A mice were used as a model of ALS (Jackson Laboratory, Bar Harbor, ME, USA). The hemizygous G93A mice were maintained by mating transgenic males with wild-type (WT) females. G93A and WT mice were housed under standard conditions (temperature 22°C, relative humidity 60%, 12 h light/dark cycles, and free access to food and water) in the animal facility at the School of Pharmacy, Nihon University. Genotyping was performed using genomic DNA extracted from tails and analysed by polymerase chain reaction (PCR) as reported previously [17]. We used a total of 60 mice, either G93A or WT mice, allocated to the following two groups: 19/60 were used for survival analyses, and the remaining 41/60 mice were used for biochemical and histological studies (Figure 1). All efforts were made to minimise the number of animals used and their distress. All experiments with animals complied with the Guidelines for Animal Experiments at Nihon University.

### 2.2. MBP Treatment Trial

MBP, the brand name Musashino Miyako BP® (MMBP®), was obtained as a generous gift from Musashino Research Institute for Immunity (Miyako Island, Okinawa, Japan) [11]. MBP was dissolved in injection water, and a fresh solution was prepared daily. Male G93A mice were randomly divided into MBP-treated and vehicle control groups; each animal in the treatment group had a littermate in the vehicle group (Figure 1). Beginning at 105 days old (15 weeks old), G93A mice were treated with either the vehicle (injection water from the Japanese Pharmacopoeia) or MBP at a dose of 2 g/kg/day oral gavage administration using a disposable oral gavage syringe (Fuchigami, Kurume, Japan) on weekday (5 days a week) mornings.

### 2.3. Motor Performance and End Point (Clinical Assessment)

Mouse motor performance was evaluated weekly using a rotarod apparatus (Muromachi Kikai, Tokyo, Japan), as described previously [17]. After the training period of 14 days, mice were able to stay on the rotarod rotating at a speed of 24 revolutions per minute (rpm). The maximum allowable score was 300 s, and the average time of three trials for each mouse was recorded twice a week. The observers were blinded with regard to treatment by MBP but performed their assessment concurrently. The end-point was defined as the inability of the mouse to right itself within 30 s after being placed on its side [2]. At that point, mice were euthanatised with CO_2_.

### 2.4. Western Blotting

Western blots were performed as reported previously [17, 18]. Spinal cord tissue obtained from 18-week-old G93A and WT mice were homogenised in radio-immunoprecipitation assay (RIPA) buffer containing 150 mM NaCl, 1% Nonidet P-40, 0.5% sodium deoxycholate, 0.1% sodium dodecyl sulfate (SDS), 50 mM Tris-HCl (pH 8.0), 1% Triton X-100, and 5 mM EDTA. The homogenate was centrifuged, and the supernatant was collected and used for downstream analyses. Protein concentrations were determined using the method of Bradford. Protein extracts were separated by SDS-polyacrylamide gel electrophoresis and transferred onto polyvinylidene difluoride (PVDF) membranes (Millipore, Billerica, MA, USA). The membranes were blocked in blocking buffer containing 20 mM Tris-HCl (pH 7.6), 137 mM NaCl, 0.05% Tween-20, and 5% skimmed milk for 1 h at room temperature (25°C) and incubated with anti-nonphosphorylated neurofilament (SMI32) antibody (SMI-32R, Millipore, Billerica, MA, USA; diluted at 1 : 2000), anti-Iba-1 antibody (016-20001, Wako, Osaka, Japan; diluted at 1 : 500), anti-glial fibrillary acidic protein (GFAP) antibody (MAB360, Millipore, Billerica, MA, USA; diluted at 1 : 1000), or rabbit polyclonal anti-SOD1 antibody (sc-11407, Santa Cruz Biotechnology Inc., Santa Cruz, CA, USA; diluted at 1 : 2000) overnight at 4°C. The membranes were washed repeatedly in Tris-buffered saline (20 mM Tris-HCl pH 7.6, 137 mM NaCl) containing 0.05% Tween-20 and incubated with horseradish peroxidase- (HRP-) conjugated secondary antibody (Santa Cruz Biotechnology Inc., Santa Cruz, USA; diluted at 1 : 20000) for 1 h. Immunoreactive bands were detected using an enhanced chemiluminescence (ECL) detection system (GE Healthcare Biosciences, UK). The optical density of the bands detected on the blots was measured using Scion imaging software (Scion, Frederick, MD, USA). Quantitative results were expressed as the ratio of the band intensity of the protein of interest to the band intensity of *β*-actin (A5441, Sigma-Aldrich, St. Louis, MO, USA; diluted at 1 : 2000).

### 2.5. Immunohistochemistry

Immunohistochemistry was performed as described elsewhere [19, 20]. Briefly, anaesthetised animals were perfused with 4% paraformaldehyde in phosphate-buffered saline (PBS). Postfixed lumbar spinal cords were horizontally sectioned on a cryostat at a thickness of 20 *μ*m. After blocking nonspecific binding by incubating with 1.5% normal goat serum in 0.1% Triton X-100/PBS, the sections were incubated anti-Iba-1-antibody (019-19741, Wako, Osaka, Japan, diluted at 1 : 500) or anti-GFAP Alexa Fluor 488-conjugated antibody (53-9892, eBioscience, San Diego, CA, USA; diluted at 1 : 2000) for 48 h at 4°C. After washing with PBS, the sections labelled with the anti-Iba-1 antibody were incubated with Alexa Fluor 488-conjugated rabbit IgG secondary antibody (A21206, Thermo Fischer Scientific, San Diego, CA, USA; diluted at 1 : 1000) for 2 h. After rinsing with PBS, the sections were analysed using a confocal laser microscope (LSM-710, Zeiss, Oberkochen, Germany). Semiquantitative analysis of change in GFAP and Iba-1 immunoreactivity was performed as reported previously [16].

### 2.6. Histological Analysis

Cresyl violet stain was performed as described elsewhere [21]. Postfixed lumbar spinal cords were horizontally sectioned on a cryostat at a thickness of 20 *μ*m. The paraffin-embedded spinal cord sections were stained with cresyl violet (Sigma-Aldrich, St. Louis, MO, USA). Images were collected with an inverted microscope (IX71; Olympus Co., Tokyo, Japan). A blinded observer counted the number of motor neurons in the anterior grey matter (left or right) with the aid of image processing software (ImageJ, National Institutes of Health, Bethesda, MD, USA). Motor neurons were defined according to the following three criteria: (i) Nissl-stained cell, (ii) localisation in ventral horns, and (iii) diameter > 25 *μ*m.

### 2.7. Statistics

All data were expressed as the mean ± standard error of the mean (SEM) or standard deviation (SD). Serial changes in motor performance were analysed with two-way repeated measure analysis of variance (ANOVA) (with “drug treatment” and “weeks of age” as between-subjects' factors) followed by Bonferroni's *post hoc* test. The survival data were analysed using the Kaplan-Meier with the Mantel-Cox log-rank test. Expression levels of protein and quantification of motor neuron number were analysed using one-way repeated measure ANOVA followed by Tukey's *post hoc* test. Expression levels of SOD1 protein were compared using Student's *t*-test. Semiquantitative *p* values of <0.05 indicated statistical significance.

## 3. Results

### 3.1. MBP Extended the Survival and Improved the Motor Performance in G93A Mice

**Figure 2:**
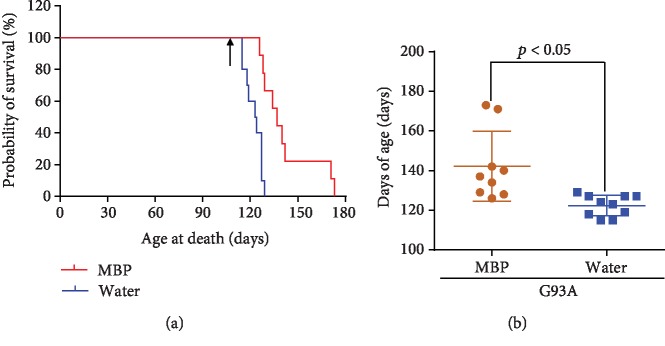
Effect of MBP on the survival of G93A mice. Mice were orally administered with injection water (vehicle) or MBP, starting at a late symptomatic stage (15 weeks old). (a) Survival curve of G93A mice treated with vehicle or MBP, analysed by Kaplan-Meier analysis with the Mantel-Cox log-rank test (*n* = 9-10, *p* = 0.004). The arrow indicates the start of vehicle or MBP administration. (b) The graph shows the maximum lifespan of G93A mice treated with vehicle or MBP. Values represent the mean ± SD. Statistical significance was determined by unpaired Student's *t*-test. (*n* = 9-10).

Starting at 105 days old (15 weeks old), male G93A mice were treated orally with 2 g/kg/day MBP or injection water (vehicle) on weekdays (5 days a week). Mice received continuous treatment until the end stages of the disease. Treatment with MBP significantly prolonged the survival of G93A mice. The median survival of vehicle-treated G93A mice was 123.5 days (*n* = 10), whereas treatment with MBP increased the lifespan of G93A mice to 137.0 days (*n* = 9), with an increase of 13.5 days (Figure 2(a)). The survival curve of MBP-treated mice was compared to that of vehicle-treated G93A mice using the Mantel-Cox log rank test, and the significant difference was found between the two curves (*p* = 0.004). Moreover, oral administration of MBP significantly increased the mean survival of G93A mice by approximately 20 days from 122 days to 142 days (Figure 2(b)).

**Figure 3:**
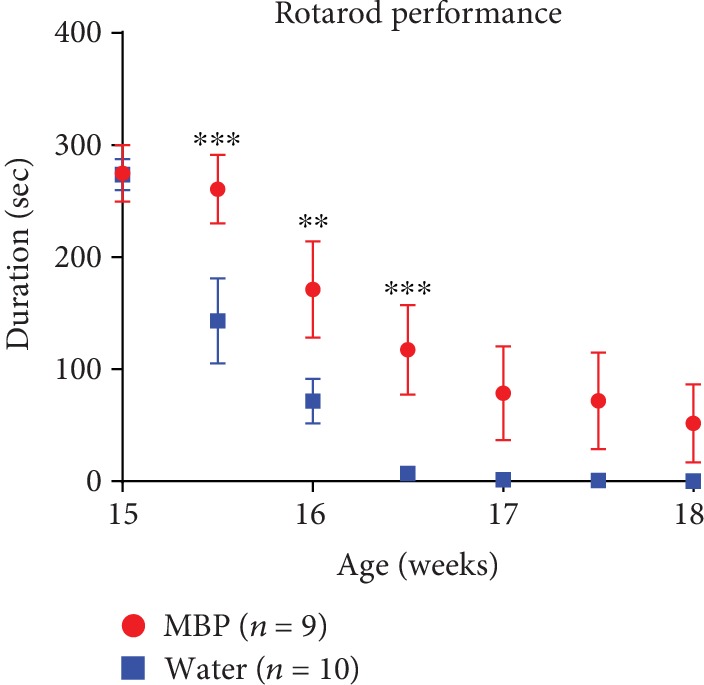
Effect of MBP on the motor performance of G93A mice. Mice were orally with administered injection water (vehicle) or MBP, starting at a late symptomatic stage (15 weeks old). Motor performance of the mice was evaluated using a rotarod apparatus. The graph depicts latency to fall from the rotarod apparatus in G93A mice treated with vehicle or MBP. Values represent the mean ± SEM. Serial changes in motor performance were analysed with two-way ANOVA (with “drug treatment” and “weeks of age” as between-subjects' factors) followed by Bonferroni's *post hoc* test (*n* = 9-10). ^∗∗∗^*p* < 0.001 and ^∗∗^*p* < 0.01 vs. aged-matched mice treated with vehicle.

When untreated 15-week-old mice were assessed on an accelerating rotarod starting, all mice displayed the maximum allowable score (300 s) in latency to fall from the rod. In agreement with our previous results [17], vehicle-treated G93A mice developed hind limb weakness including reduced running time on the rotarod apparatus at 15.5 weeks and beyond (Figure 3). At 17 weeks, all vehicle-treated G93A mice showed paralysis and no motor performance was possible. In contrast, MBP-treated G93A mice showed a significantly longer duration of motor performance than vehicle-treated mice (Figure 3). Importantly, between 15.5 weeks and 16.5 weeks of age, there was a significant improvement in motor performance in MBP-treated G93A mice compared to that of vehicle-treated G93A mice (Figure 3).

### 3.2. MBP Decreased Motor Neuron Loss in the Spinal Cord of G93A Mice

**Figure 4:**
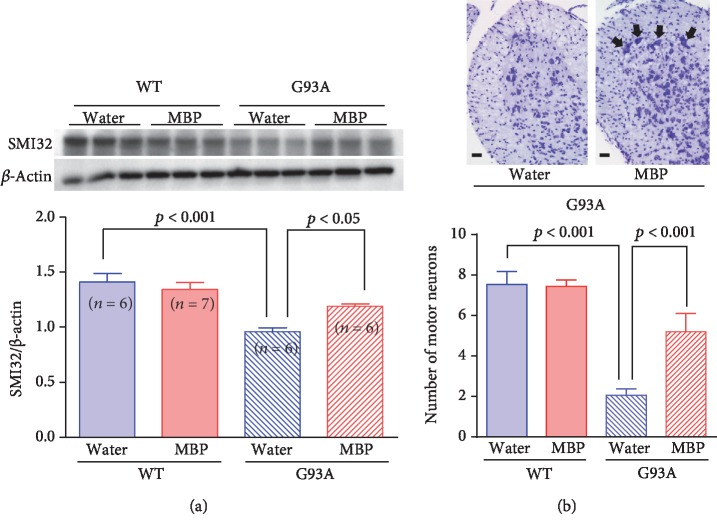
MBP ameliorates motor neuron loss in the spinal cord of G93A mice. Mice were orally administered with injection water (vehicle) or MBP, starting at a late symptomatic stage (15 weeks old). Three weeks after the start of the treatment, the lumbar spinal cords were analysed by western blot and the histopathology was analysed. (a) Photographs show representative western blots of SMI32, a marker of motor neurons, in the lumbar spinal cord of male G93A mice and WT mice. Equal amounts of cell lysates (10 *μ*g) were analysed, with *β*-actin as an internal control. The graph shows the relative densities of each band on the blots estimated quantitatively using Scion imaging software. Quantitative data are expressed as the ratio of the band intensity of SMI32 to the band intensity of *β*-actin. Each value represents the mean ± SD. Statistical significance was determined by using one-way ANOVA followed by Tukey's *post hoc* test (*n* = 6-7). (b) Photographs show representative cresyl violet-stained sections of the lumbar spinal cord in the indicated groups of mice at 18 weeks old. Arrows indicate motor neurons. Scale bar indicates 100 *μ*m. The graph shows the number of surviving motor neurons in lumbar spinal cord sections from the indicated groups of mice. Values represent the mean ± SEM. Statistical significance was determined by using one-way ANOVA followed by Tukey's *post hoc* test (*n* = 4).

To determine whether the therapeutic potential of MBP was attributable to the suppression of spinal motor neuron degeneration, we evaluated the number of motor neurons in the spinal cord. Three weeks after the start of the treatment with vehicle or MBP, the lumbar spinal cord lysates were prepared and analysed by western blot. Although the protein levels of SMI32, a marker of motor neurons [17], significantly decreased in the spinal cord of G93A mice compared to those in WT mice, this reduction in SMI32 levels was alleviated upon MBP treatment (Figure 4(a)). Conversely, MBP had no effect on the expression level of SMI32 in the spinal cord of WT mice (Figure 4(a)).

Next, we assessed the number of motor neurons remaining in the lumbar spinal cord of G93A mice by Nissl staining. The micrograph of cresyl violet-stained lumbar spinal cord sections from vehicle-treated G93A mice showed that a large number of the motor neurons in the ventral horn was lost at 18 weeks of age and that vacuolisation was apparent in the ventral horn in the lumbar segment (Figure 4(b)). Consistent with the preservation of SMI32 expression, the loss of motor neurons in the spinal cord also reduced significantly after treatment with MBP for 3 weeks. In WT mice, neurodegeneration was not observed in the spinal cord at any age (Figure 4(b)).

### 3.3. MBP Alleviated Astrocytosis and Microglial Activation in the Spinal Cords of G93A Mice

**Figure 5:**
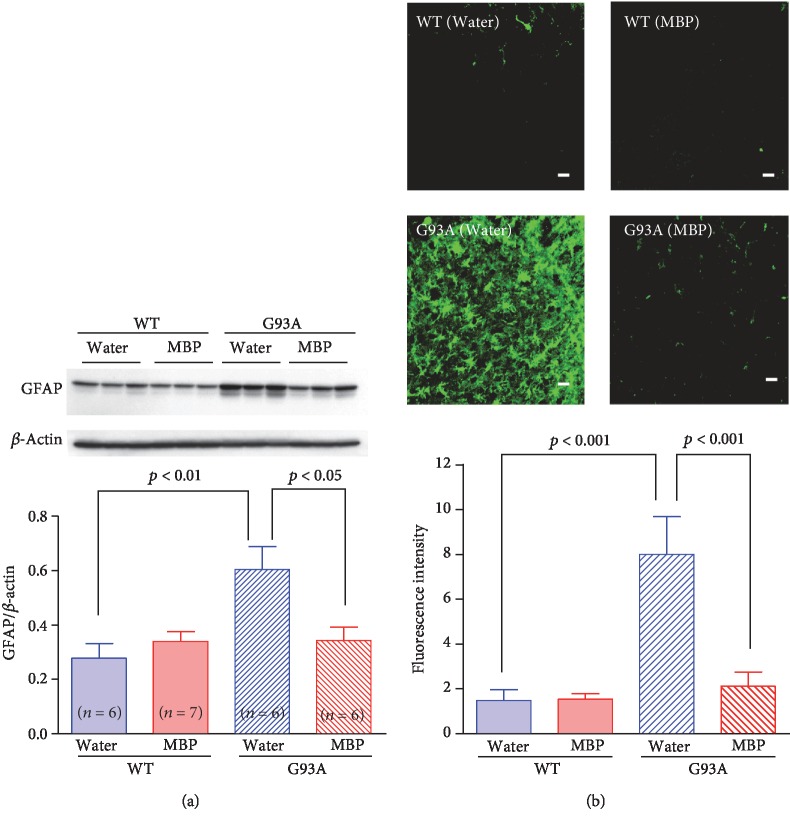
MBP attenuates morphological changes in astrocytes in G93A mice. Mice were orally administered with injection water (vehicle) or MBP, starting at a late symptomatic stage (15 weeks old). Three weeks after the start of the treatment, the lumbar spinal cords were analysed by western blot and the histopathology was imaged. (a) Photographs depict a representative western blot of GFAP, an astrocyte marker, in the lumbar spinal cord of male G93A mice and WT mice. Equal amounts of cell lysates (10 *μ*g) were analysed, with *β*-actin as an internal marker. The graph shows the relative density of each band on the blots estimated quantitatively using Scion imaging software. Quantitative data are expressed as the ratio of the band intensity of GFAP to the band intensity of *β*-actin. Each value represents the mean ± SD. Statistical significance was determined by using one-way ANOVA followed by Tukey's *post hoc* test (*n* = 6-7). (b) Photographs show representative confocal images of immunofluorescence staining for GFAP in the lumbar spinal cord sections from the indicated groups of mice at 18 weeks old. Representative data from four separate experiments are presented. Scale bar indicates 20 *μ*m. The graph shows semiquantitative analysis of changes in GFAP immunoreactivity in motor neurons. The fluorescence intensity of GFAP immunoreactivity was analysed quantitatively using Scion imaging software. Values represent the mean ± SEM. Statistical significance was determined by using one-way ANOVA followed by Tukey's *post hoc* test (*n* = 4).

**Figure 6:**
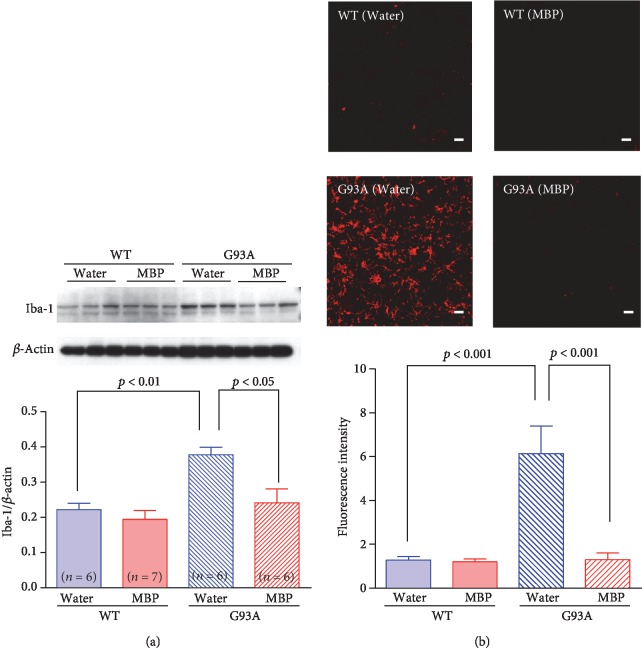
MBP attenuates morphological changes in microglia in G93A mice. Mice were orally with administered injection water (vehicle) or MBP, starting at a late symptomatic stage (15 weeks old). Three weeks after the start of the treatment, the lumbar spinal cords were analysed by western blot and histopathology was analysed. (a) Photographs show representative western blots of Iba-1, a microglia marker, in the lumbar spinal cord of male G93A mice and WT mice. Equal amounts of cell lysates (10 *μ*g) were analysed, with *β*-actin as an internal marker. The graph shows the relative density of bands on the blots estimated quantitatively using Scion imaging software. Quantitative data are expressed as the ratio of the band intensity of Iba-1 relative to the band intensity of *β*-actin. Each value represents the mean ± SD. Statistical significance was determined by using one-way ANOVA followed by Tukey's *post hoc* test (*n* = 6-7). (b) Photographs show representative confocal images of immunofluorescence staining for Iba-1 in the lumbar spinal cord sections from the indicated groups of mice at 18 weeks old. Representative data from four separate experiments are presented. Scale bar indicates 20 *μ*m. The graph shows semiquantitative analysis of changes in Iba-1 immunoreactivity in motor neurons. The fluorescence intensity of Iba-1 immunoreactivity was analysed quantitatively using Scion imaging software. Values represent the mean ± SEM. Statistical significance was determined by using one-way ANOVA followed by Tukey's *post hoc* test (*n* = 4).

It is generally accepted that motor neuron degeneration is accompanied by the activation of glial cells in the ALS mouse model [22]. To further characterise the effects of MBP on the activation of glial cells, both WT mice and G93A mice were sacrificed after 3 weeks of treatment to evaluate GFAP (Figure 5) and Iba-1 (Figure 6) immunoreactivity in the spinal cord as indicators of astrogliosis and microglial activation, respectively. Western blotting revealed that the protein expression levels of GFAP and Iba-1 in G93A mice were significantly higher than those of WT mice at the end stage of the disease. Oral MBP treatment significantly suppressed the elevated protein levels of GFAP and Iba-1 observed in G93A mice. Likewise, GFAP and Iba-1 immunoreactivity was hardly detected in the anterior horn of WT mice, whereas these G93A mice were positive for both GFAP and Iba-1. Immunofluorescence staining indicated that activated astrocytes and microglia were abundant in vehicle-treated G93A mice. MBP treatment dramatically and significantly ameliorated the activation of astrocyte and microglia in lumbar spinal cord sections from G93A mice.

### 3.4. MBP Did Not Affect SOD1 Protein Expression in the Spinal Cords of G93A Mice

**Figure 7:**
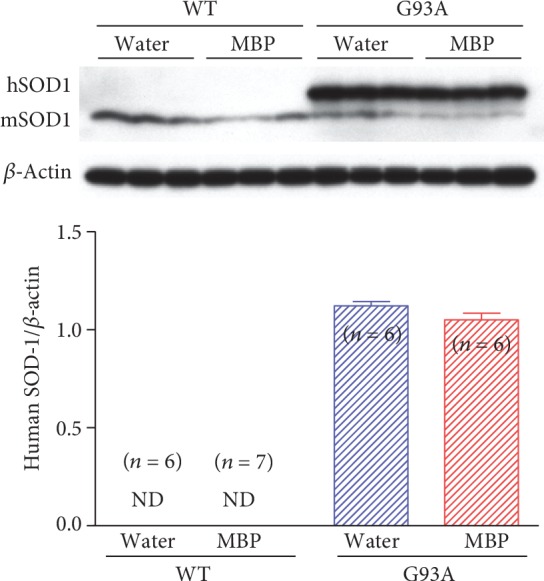
Effect of MBP on SOD1 protein expression in spinal cord tissue of G93A and WT mice. Mice were orally administered with injection water (vehicle) or MBP, starting at a late symptomatic stage (15 weeks old). Three weeks after the start of the treatment, the lumbar spinal cords were analysed by western blot. Photographs show representative western blot of SOD1 in the lumbar spinal cord of male G93A mice and WT mice. The upper band represents human SOD1 (hSOD1; 21 kDa) and the lower band represents mouse SOD1 (mSOD1; 16 kDa). Equal amounts of cell lysates (10 *μ*g) were analysed, with *β*-actin as an internal marker. The graph shows the relative density of bands on the blots estimated quantitatively using Scion imaging software. Quantitative data are expressed as the ratio of the band intensity of hSOD1 relative to the band intensity of *β*-actin. Each value represents the mean ± SD. Statistical significance was determined by unpaired Student's *t*-test (*n* = 6-7). ND: not detected.

Using protein homogenates of spinal cords from G93A mice and age-matched WT mice, we analysed the expression of endogenous mouse SOD1 (mSOD1) and mutant human SOD1 (hSOD1) protein. As shown in Figure 7, the examination of protein expression by western blot revealed the presence of endogenous mSOD1 in the lumber spinal cord of both WT and G93A mice at the end stage of the disease. While no bands corresponding to mutant hSOD1 were noted in spinal cord extracts of WT mice, hSOD1 was observed in the lysates of G93A mice (Figure 7). At the end stage of the disease, the protein expression of the mutant hSOD1 did not change in G93A mice with MBP treatment and remained at a level similar to that detected in vehicle-treated mice.

## 4. Discussion

ALS is a progressive and lethal degenerative disease of motor neurons. At present, there are only two approved drugs, both of which are poorly effective for the treatment of ALS. Therefore, there is an increased need to develop new therapies to cure and/or ameliorate the severe course of the disease. In this study, we determined that oral administration of MBP, immediately after the onset of ALS-like symptoms, delayed the deterioration of motor function and extended survival duration in G93A mice. We also show that these improvements were associated with a reduction in reactive astrocytes and activated microglial cells and delayed motor neuron loss in the spinal cord. Overall, our results clearly show that the oral administration of MBP after ALS symptom onset can slow disease progression and that MBP is a potential therapeutic agent for the treatment of ALS.

MBP, as well as BP, is generally used as ethnomedicine and functional food worldwide. In humans, BP extract administered orally at a dose of 400 mg/kg of body weight for 3 months had no noticeable toxicity [23]. Moreover, a BP dose of 27 g/kg body weight has been shown to confer antiobesity and antidiabetic effects without any obvious signs of toxicity on leptin-deficient (ob/ob) mice [24]. In this study, we observed that both WT and G93A mice did not show any toxicity relevant to the spinal cord tissue (Figures 4–6) and any abnormal behavior (data not shown) following a daily dose of MBP at 2 g/kg body weight. These results suggest that the oral use of MBP or BP may not be toxic and is potentially safe. In contrast, a number of polyphenols isolated from MBP are described as active components involved in suppressing oxidative stress, inflammation, and allergy *in vitro* and *in vivo* [11–13, 15, 16]. Among the components of MBP, caffeic acid, chlorogenic acid, and quercetin have been reported to reduce oxidative stress and increase the viability of NSC34 cells, a motor neuron-like cell line, expressing mutant *SOD1*^G93A^ linked to human ALS [25]. Furthermore, the anti-inflammatory activity of chlorogenic acid [26], quercetin [27], and rutin [28] has been shown to ameliorate spinal cord injury. Therefore, it is possible that the various active components present in MBP interact with each other, to produce a synergistic neuronal protective effect in the spinal cord of G93A mice.

To date, many of the proposed therapeutic approaches used in G93A mice administer treatment before the onset of ALS symptoms. As most cases of ALS are sporadic, pre-symptomatic assessment is impossible in these patients. Thus, an effective model to study the behavior of ALS observed in the majority of ALS patients has not been devised. Although edaravone and riluzole are currently available for ALS treatment, post symptomatic administration provides only limited effects on survival [29, 30]. In fact, riluzole treatment significantly prolonged the survival of G93A mice by 7.5% compared to that of untreated G93A mice (untreated G93A mice: 126.1 days vs. riluzole-treated G93A mice: 135.5 days); however, treatment was initiated in 30-day-old mice [31]. In contrast, when riluzole was administered to 100-day-old (14-weeks-old) G93A mice, no beneficial effects were observed and treatment did not extend survival relative to untreated G93A mice (delayed for 3.0 days) [32]. Similarly, in patients with ALS, riluzole has poor efficacy during the later stages of the disease [33]. In addition, it has been reported that treatment with edaravone, initiated after the onset of ALS symptoms, does not improve survival of G93A mice (delayed for 2.2 days) [34]. In this study, we demonstrated that oral MBP treatment, beginning after the onset of ALS symptoms, significantly improved the deterioration of motor performance and prolonged the survival (16.4%) of G93A mice (untreated G93A mice: 122 days vs. MBP-treated G93A mice: 142 days). Therefore, the efficacy of MBP was approximately twice that of riluzole, and it drastically improved survival in G93A mice unlike edaravone and riluzole. The present findings provide a new insight into MBP activity that may be applicable when considering therapeutic options for not only fALS but also the sporadic type. Further studies will be necessary to evaluate the efficacy of MBP in combination with riluzole or edaravone for the treatment of ALS.

It has been reported that traditional Chinese medicine might be beneficial to prolong the survival of ALS model mice. *Hirsutella sinensis* significantly extended the lifespan of G93A mice by approximately 17 days from 127 days to 144 days [35]. Moreover, *Huolingshengji Formula* which consists of six herbs including *Epimedium Herb*, *Radix Astragali*, *Fructus Corni*, *Radix Rehmanniae*, *Poria cocos*, and *Atractylodes macrocephala Koidz* prolongs the lifespan of G93A mice by approximately 11 days from 130 days to 141 days [36]. However, the administration of those traditional medicines was initiated from a presymptomatic stage of G93A mice. Interestingly, treatment with *Huolingshengji* from the day of disease onset prolongs the lifespan of G93A mice by approximately 8 days from 130 days to 138 days [36]. Our results showed that MBP significantly increases the mean survival of G93A mice by approximately 20 days. These results suggest that MBP is a beneficial medicine for ALS compared with other traditional medicines.

Neuroinflammation is the activation of an immune response in the central nervous system by activated astrocytes and microglial cells. Activation of astrocytes and microglia is prominently observed in regions of degenerating motor neurons in ALS patients as well as in model mice [4, 5, 37, 38]. A previous study conducted in our laboratory showed that the levels of spinal GFAP and Iba-1 expression were elevated in an age-dependent manner in G93A mice [17]. The increase in the spinal expression of GFAP in G93A mice progressed more slowly than that of Iba-1, but both levels were significantly higher at the end stages of the disease (17 and 19 weeks old) than those in age-matched WT mice [17]. In this study, western blotting analysis also showed that GFAP and Iba-1 immunoreactivity in the spinal cord of G93A mice dramatically increased at 18 weeks relative to age-matched WT mice. Activated astrocytes and microglia can induce neuronal death by exerting inflammatory effector functions. For example, astrocyte activation in ALS is associated with a decrease in the expression of glutamate transporters [39], increased levels of ROS and inducible nitric oxide synthase [40], and elevated production of proinflammatory cytokines, such as interferon-*γ* [41] and transforming growth factor-*β* [42]. Moreover, activated microglia secreted proinflammatory cytokines and oxidative stress mediators including hydrogen peroxide and nitric oxide and play pivotal roles in the pathogenesis of ALS [43, 44]. Therefore, the pharmacological approach targeting neuroinflammation induced by activated astrocyte and microglia is promising for the development of therapeutic strategies for ALS.

We demonstrated for the first time that MBP treatment markedly suppresses activation of microglia and astrocytes in the spinal cords of G93A mice. Anti-inflammatory effects have been found not only for MBP but also for BP in a variety of animal models [15, 16, 45]. Further studies are required to clarify the anti-inflammatory roles of MBP in the spinal cords of G93A mice. A previous study has reported that the antioxidant active compounds including caffeic acid, six kinds of chlorogenic acids, and seven kinds of flavonoids are identified as the constituents of MBP [12]. However, our results provide a strong evidence that attenuation of the activation of astrocytes and microglia is closely linked to the efficacy of MBP in G93A mice. Reactive microglia and astrocytes have been identified in the spinal cords isolated from patients with sporadic ALS [37, 38]; therefore, MBP may be useful not only for familial but also for sporadic ALS. In addition, since glial activation plays a pivotal role in the progression of various neurodegenerative diseases caused by neuroinflammation, MBP may be effective for the treatment of other neurodegenerative disorders.

## 5. Conclusions

In conclusion, this study has demonstrated for the first time that oral treatment with MBP at the onset of symptoms of neurodegeneration prolongs the life span, improves motor performance, and attenuates motor neuron loss and glial activation in fALS model mice, G93A. Further studies will be required to identify the molecular targets and mechanisms of these effects and to clarify the therapeutic potential of MBP in ALS patients and other neurodegenerative diseases. These significant preclinical findings together with the clinical safety profile of MBP support its potential application as a promising candidate drug for the therapy of fALS caused by mutant *SOD1* and possibly sporadic ALS.

## Data Availability

The data used to support the findings of this study are available from the corresponding authors upon request.
